# A Highly Efficient Approach to Protein Interactome Mapping Based on Collaborative Filtering Framework

**DOI:** 10.1038/srep07702

**Published:** 2015-01-09

**Authors:** Xin Luo, Zhuhong You, Mengchu Zhou, Shuai Li, Hareton Leung, Yunni Xia, Qingsheng Zhu

**Affiliations:** 1 X. Luo, Y. Xia and Q. Zhu are with the College of Computer Science, Chongqing University, Chongqing, 400044 China; 2 X. Luo, Z. You, S. Li and H. Leung are with the Department of Computing, Hong Kong Polytechnic University, Hong Kong, HK 999077, China; 3 M. Zhou is with the Department of Electrical and Computer Engineering, New Jersey Institute of Technology, Newark, NJ 07102, USA

## Abstract

The comprehensive mapping of protein-protein interactions (PPIs) is highly desired for one to gain deep insights into both fundamental cell biology processes and the pathology of diseases. Finely-set small-scale experiments are not only very expensive but also inefficient to identify numerous interactomes despite their high accuracy. High-throughput screening techniques enable efficient identification of PPIs; yet the desire to further extract useful knowledge from these data leads to the problem of binary interactome mapping. Network topology-based approaches prove to be highly efficient in addressing this problem; however, their performance deteriorates significantly on sparse putative PPI networks. Motivated by the success of collaborative filtering (CF)-based approaches to the problem of personalized-recommendation on large, sparse rating matrices, this work aims at implementing a highly efficient CF-based approach to binary interactome mapping. To achieve this, we first propose a CF framework for it. Under this framework, we model the given data into an interactome weight matrix, where the feature-vectors of involved proteins are extracted. With them, we design the rescaled cosine coefficient to model the inter-neighborhood similarity among involved proteins, for taking the mapping process. Experimental results on three large, sparse datasets demonstrate that the proposed approach outperforms several sophisticated topology-based approaches significantly.

Protein-protein interactions (PPIs), or known as protein interactomes, are very important in various biological processes and form the basis of biological mechanisms. During the last decade, the progress of high-throughput screening (HTS) techniques, e.g., canonical yeast two-hybrid assay^1^, tandem affinity purification and mass spectrometric^2^, mass spectrometric protein complex identification^3^, and protein fragment complementation^4^, has resulted in rapid accumulation of data describing global networks of PPIs in organisms^1^. Several HTS-PPI datasets were published for various organisms, such as humans (Homo sapiens)^5^, worms (Caenorhabditis elegans)^6^, yeast (Saccharomyces cerevisiae)^7^, fly (Drosophila melanogaster)^8^, and plants^9^. With these obtained HTS-PPI data, great opportunities in studying biological events are unprecedented.

Initially, due to the limitations of experimental techniques, HTS-PPI data are prone to high rate of false-positives, i.e., HTS-PPIs identified by the experiments do not actually exist in nature^10^,^11^. With the advance of related technology, the quality of HTS-PPI data is greatly improved in recent years^12^,^13^,^14^. Nonetheless, HTS techniques have not yet reached the perfection and false-positive noises can still be found in their output^12^,^13^,^14^. Meanwhile, in spite of their efficiency, it is still very hard for HTS methods to identify the full PPI network of given species^10^,^11^. Hence, the obtained HTS-PPI data cannot cover all potential PPIs either.

Although HTS-PPI data have made advances to identify the PPI networks, it is desired to extract more useful knowledge from them. Various efforts have been made to do so^15^,^16^,^17^,^18^,^19^,^20^,^21^,^22^, e.g., solving the problem of binary interactome mapping (BIM). The main BIM task is to analyze the obtained HTS-PPIs to address the following two issues^15^,^16^,^17^,^18^,^19^,^20^,^21^,^22^,

- Assessment: assessing the reliability of obtained HTS-PPI data, and rejecting the unreliable interactomes to decrease their false-positive rate; and
- Prediction: predicting the probable interactomes suggested by the obtained HTS-PPIs.

Among current approaches to the problem of BIM, network topology-based methods^23^,^24^,^25^,^26^,^27^ have proven to be efficient. Their main idea is to address the BIM problem by analyzing solely the topology of the network corresponding to given HTS-PPI data^23^,^24^,^25^,^26^,^27^, thereby requiring no prior knowledge of individual proteins.

Saito *et al.*^23^ rank the reliability of HTS-PPIs by the interaction generality (IG) extracted from the local topology of each protein-pair. Brun *et al.*^24^ employ Czekanowski-Dice distance (CD) to analyze the neighborhood topology of each protein for classification tasks. Chen *et al*.^25^ propose the interaction reliability by alternative path (IRAP), which models the protein-protein relationship through exploring the path information of an HTS-PPI network. Chua *et al.*^26^,^27^ propose the functional similarity weight (FW), which is highly efficient in representing the relationship among proteins based on HTS-PPI data. As indicated in^26^,^27^, FW is able to outperform IG, CD and IRAP in addressing the BIM problem in many circumstances.

Topology-based approaches take advantage in purely relying on HTS-PPI data without requiring any additional information of proteins^23^,^24^,^25^,^26^,^27^. However, they share the drawback of low efficiency on sparse HTS-PPI networks, which are very common in real applications^15^,^16^,^17^,^18^,^19^,^20^,^21^,^22^. For instance, the HTS-PPI network of the IntAct dataset^28^ contains 13,807 HTS-PPIs among 4,486 proteins; the average degree on each protein is about 3.08, and the network density is 0.14% only. On such a sparse network, the performance of topology-based methods tends to deteriorate significantly^26^.

To address the BIM problem on large, sparse HTS-PPI networks, this work aims at developing a highly-efficient approach to BIM based on collaborative filtering (CF). CF is initially designed for the problem of personalized-recommendation in e-commerce^29^,^30^,^31^,^32^. Such a problem generally involves three fundamental kinds of entities, i.e., users, items (e.g., movies and news), and user-item usage history (e.g., scores and comments). The main issue is to figure out useful patterns reflecting the connection between users and items from user-item usage history, and then make reliable predictions for possible user-item links according to these patterns^29^,^30^,^31^,^32^. Since each user can only contact a tiny fraction of the whole item set, known user-item pairs are far less than unknown ones. In other words, the problem of personalized-recommendation features with sparsity, and CF-based approaches have proven to be very effective in dealing with it^29^,^30^,^31^,^32^.

Through careful investigations of these two problems, i.e., BIM and personalized-recommendation, we find that their solution spaces are very similar: the key to both problems is to model the relationship among involved entities based on incomplete information. Motivated by this intuition, we propose a novel CF-based approach to the BIM problem, thereby resulting in a new class of methods for such problems. According to our best knowledge, such efforts have been never seen in any previous work. The main contributions of this work include:

- A CF framework for the BIM problem, which is a novel computational paradigm for such kind of problems;
- A novel approach to the BIM problem in context of binary HTS-PPI data based on the CF framework;
- Rescaled cosine coefficient (RCC), a novel metric able to accurately model the protein-protein relationship corresponding to the given HTS-PPI data; and
- Empirical validations of the proposed concepts and framework via two public large, real datasets.

## Results

### Methods for Comparison

This work considers the cases where only binary HTS-PPI data are available. The proposed RCC-based CF (RCF) approach to BIM is highly flexible, and is able to work depending on binary HTS-PPI data solely. Therefore, it is fair and reasonable to compare the proposed RCF against sophisticated topology-based methods, which are well known for their efficiency and dependence on HTS-PPI data only. Three topology-based algorithms, which respectively employ IG, CD and FW as the indexing metric, are implemented and compared against RCF.

### Datasets

Three public large, real datasets are the Homo sapiens protein interaction data from the IntAct database^28^, the BioGrid database^33^, and the human signaling dataset by Wang's Lab^34^,^35^,^36^,^37^. Their details are listed below.

- D1: the IntAct dataset consisting of 4,486 proteins and 13,807 Homo sapiens HTS-PPIs^28^, where the average degree on each protein is about 3.08, and the density of the corresponding HTS-PPI network is 0.14% only; and
- D2: the Homo sapiens HTS-PPI dataset from the BioGrid database^33^. D2 contains 7,493 proteins and 27,045 HTS-PPIs. Its average degree on each protein is about 3.61, and the density of the corresponding HTS-PPI network is 0.10% only.Note that both the datasets correspond to very sparse HTS-PPI networks. About 99.9% entries in the corresponding IW matrices are unknown.
- D3: the physical links from the human signaling network that is manually curated by Wang's Lab^34^,^35^,^36^,^37^. This is the largest manually curated human signaling network, which contains more than 6,000 proteins and 63,000 relations. In this work, we employ its 21,579 physical links on 2,767 proteins, which form a network with the density of 0.28%. Note that different from D1 and D2, the PPIs in D3 are manually curated with high accuracy^34^,^35^,^36^,^37^. Hence, by using it we expect to examine different performance aspects of the proposed and other concerned methods.

### Evaluation Settings

Our experiments employ Gene Ontology (GO) based annotations to evaluate involved methods. GO is one of the most important ontologies inside the bioinformatics community^38^. Its organizing principles are cellular component, biological process, and molecular function. During our experiments, we employ them as the ground-truth to validate the performance of tested methods; such experimental designs are based on the strategy of ‘guilt by association'^39^ which provides the evidence that interactive proteins probably possess functional similarity and cellular co-localization, and are commonly accepted by related works^23^,^24^,^25^,^26^,^27^,^40^,^41^. All tested algorithms share the following experimental process:

- Assessment. On either dataset, we firstly apply each tested algorithm to evaluate the likelihood of given HTS-PPIs. Thereafter, we evaluate the functional homogeneity and localization coherence of the assessment by computing the rate of interacting protein pairs with functional roles or cellular localization in common. This rate should be high on HTS-PPIs corresponding to high likelihood.
- Prediction. On either dataset, we select the 20,000 missing interactomes corresponding to the highest likelihoods provided by each test algorithm, and then evaluate the functional homogeneity and localization coherence of the prediction by computing the rate of interacting protein pairs with functional roles or cellular localization in common. This rate should also be high on missing interactomes corresponding to high likelihood. Note that IG suffers from low efficiency when dealing with the prediction task since it assigns identically high values on missing interactomes; besides, it is also rarely employed to predict missing interactomes in^23^,^24^,^25^,^26^,^27^. Therefore, we did not test the performance of IG in predicting missing interactomes, either.

In each test, we employ the first ontology of GO terms to identify the co-localization, and the other two ontologies of GO terms to identify the functional similarity, among involved proteins respectively. This setting was also widely employed by previous works^23^,^24^,^25^,^26^,^27^,^40^,^41^. Note that their validation protocols based on the gene expression correlations^42^, or the GO semantic analysis^43^,^44^, are also efficient to examine the performance of computational approaches to BIM. However, in this work we intend to keep consistent with the existing studies regarding the same issue in terms of validation protocols, to check whether the proposed method can achieve better performance than them.

Note that GO terms are organized hierarchically into functional subfamilies, i.e., two different GO terms may have a common parent or a common child in the hierarchy. Hence, GO terms at high levels correspond to many proteins, while those at low levels correspond to rather few. To obtain objective results, we choose GO terms at middle levels in our experiments. More specifically, we remove the top 1% annotations corresponding to most proteins from the annotation data, to validate whether each tested method can correlate with the left annotations. Note that we download the GO annotation data sets from http://www.geneontology.org.

### Result Analysis

Note that the performance of RCF relies on the hyper parameters *C_Y_* and *d* as described in the Method Section. On both datasets, we set *d* = 5 and *C_Y_* = 30 for all testing cases, which are chosen based on the parameter-sensitive tests presented in the Supplementary Section.

**Figure 1:**
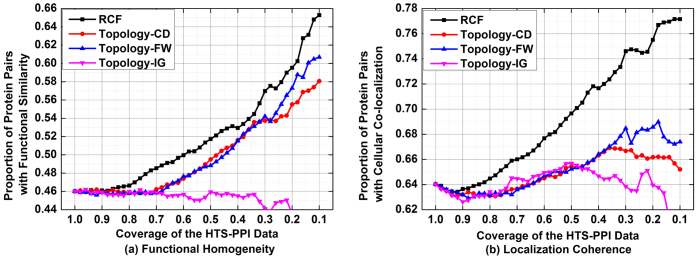
Comparison in assessing the reliability of given HTS-PPI on D1.

Figure 1 depicts the performance of all compared algorithms in HTS-PPI assessment on D1. From these results, we see that RCF obviously outperforms the tested topology-based algorithms. As shown in Figure 1(a), 51.7% of the top 50% of the HTS-PPIs ranked by RCF have a common cellular role; in contrast, topology-based algorithms can achieve 49.5% with CD, and 48.8% with FW. The proportion of interacting proteins with a common functional role hardly increases in HTS-PPI data filtered by the algorithm employing IG.

Similarly, although topology-based algorithms show high correlations with cellular co-localization on D1, RCF exhibits much better localization coherence than them. More specifically, as depicted in Figure 1(b), RCF identifies more HTS-PPIs having common cellular localization than any other algorithms do. When considering the top 50% of the filtered HTS-PPIs, 69.7% of those by RCF are supported by cellular coherence; with topology-based algorithms, this ratio drops to 65.4% by CD, and 65.2% by FW.

**Figure 2:**
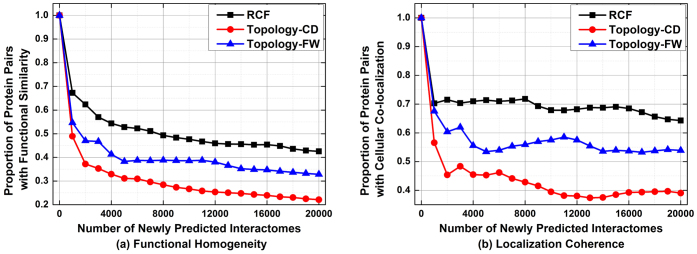
Comparison in predicting missing interactomes on D1.

Figure 2 depicts the accuracy of all tested algorithms in predicting missing interactomes on D1. From these results, we see that the prediction accuracy of RCF is clearly higher than that of the rival algorithms. For example, 42.5% of the 20,000 interactomes predicted by RCF are supported by functional similarity; with FW and CD, this ratio drops to 32.9% and 22.1%, respectively, as shown in Figure 2(a). Meanwhile, 64.3% of the 20,000 potential interactomes predicted by RCF are supported by cellular co-localizations, compared to that at 53.4% by FW, and 39.1% by CD, as shown in Fig. 2(b). During the whole comparison on D1, involved topology-based algorithms are always outperformed by RCF in terms of prediction accuracy.

**Figure 3:**
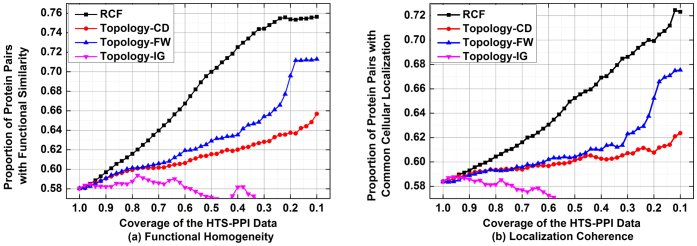
Comparison in assessing the reliability of given HTS-PPI on D2.

Figure 3 depicts the performance of involved algorithms in assessing the reliability of HTS-PPIs on D2. From these results, we see that all tested algorithms have strong correlation with functional similarity and localization coherence on D2; however, RCF performs the best. For instance, as shown in Figure 3(a), for the top 50% HTS-PPIs selected by each algorithm, 70.0% of those by RCF are supported by common functional roles; this ratio is at 61.6% by FW, 62.9% by CD, and 57.1% by IG, respectively. Meanwhile, as shown in Figure 3(b), 65.2% of the top 50% HTS-PPIs selected by RCF are supported by common cellular localizations; with FW, CD and IG, this ratio drops to 60.1%, 60.4% and 56.4%, respectively. To summarize, RCF has a clear advantage in providing steadily high efficiency when addressing the task of assessment on D2, which can be clearly observed from Figure 3.

**Figure 4:**
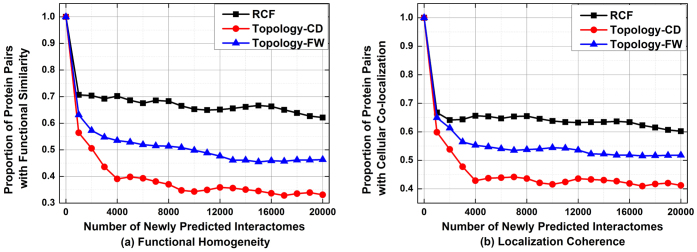
Comparison in predicting missing interactomes on D2.

Figure 4 depicts the performance of compared algorithms in predicting the missing interactomes on D2. From these results, we see that RCF is able to achieve steadily high prediction accuracy on D2. As shown in Figure 4(a), 62.1% of the 20,000 interactome predictions generated by RCF are supported by functional homogeneity; with CD and FW, this ratio is at 33.1% and 46.3%, respectively. Similar situation can be found when evaluating their correlation with cellular co-localization, as shown in Figure 4(b). When dealing with the task of prediction on D2, topology-based algorithms cannot catch up with RCF during all the tests.

**Figure 5:**
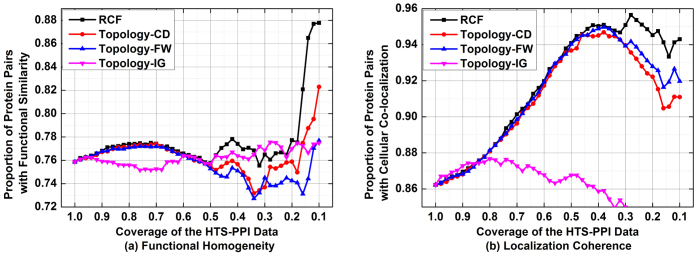
Comparison in assessing the reliability of given HTS-PPI on D3.

However, on D3, the situation is slightly different. Fig. 5 depicts the performance of involved algorithms in assessing the reliability of PPIs on D3. From this figure, we see that although RCF generally outperforms the other tested methods, it has close performance with CD and FW. For instance, as shown in Fig. 5(a), for the top 40% PPIs selected by each algorithm, 77.4% of those by RCF are supported by common functional roles; this ratio is at 75.1% by FW, 75.7% by CD, and 76.4% by IG, respectively. Meanwhile, IG can sometimes outperform the other three algorithms; e.g., for the top 30% selected PPIs, 76.9% by IG are supported by functional homogeneity in GO annotations, while RCF, FW and CD can achieve 76.5%, 73.7% and 74.5%, respectively. Similar salutations can also be found when assessing the reliability of involved PPIs with localization-coherence. As shown in Fig. 5(b), although IG cannot perform well, the performance of RCF, CD and FW are very close. For instance, 95.1% of the top 30% HTS-PPIs selected by RCF are supported by common cellular localizations; while, with FW or CD, this ratio comes to 93.9%.

**Figure 6:**
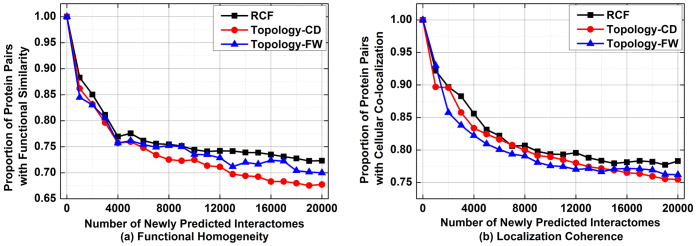
Comparison in predicting missing interactomes on D3.

Figure 6 depicts the performance of the compared algorithms in predicting the missing interactomes based on the manually curated links in D3. First of all, we notice that on this highly-accurate dataset, each tested algorithm can make good predictions sufficiently supported by GO annotations. For instance, for the 20,000 protein pairs selected by each algorithm, 78.3%, 75.5% and 76.2% of those by RCF, CD and FW are supported by functional homogeneity, respectively, as shown in Fig. 5(a). However, we also find that in terms of predicting missing interactomes, RCF can still obtain its consistent advantage in prediction accuracy when compared with the tested topology-based methods.

Based on the results on D3, we see that on highly accurate dataset like D3, RCF outperforms the rival algorithms; yet the advantage is not as obvious as that on D1 and D2. A probable reason for this phenomenon is because of the high accuracy of the manually curated PPIs in D3. After removing the top 1% of the GO annotations from the corresponding GO data, 75.9% and 86.2% of the PPIs in D3 are still supported by functional homogeneity and localization coherence, respectively. This ratio is much higher than that on D1 and D2, and suggests that few false-positive noises exist in D3. Note that as described in the last section, RCF works by controlling the impact of noises contained in the given HTS-PPI data. Hence, on dataset containing noises like D1 and D2, it can outperform the tested topology-based methods significantly. However, when the given data are highly accurate, the impact of noise data is small, and its gain is shrunk.

### Significance Tests

Based on the experimental results, we draw significance tests to validate the improvement by RCF over the state-of-the-art topology-based methods statistically. We choose to conduct the Friedman test^45^, which is effective for validating the performance of multiple methods on multiple datasets. Let 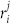 be the rank of the *j*th one of *k* algorithms on the *i*th one of *N* testing cases. The Friedman test compares the average ranks of the algorithms, 
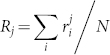
. Under the null-hypothesis, which states that all the algorithms are equivalent and so their ranks *R_j_* should be equal, the Friedman value is computed as^45^:
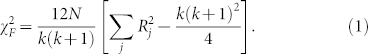
With (1), the test score is given by
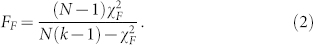
Note that (2) is distributed according to the *F*-distribution with *k*-1 and (*k*-1)(*N*-1) degrees of freedom^45^. Hence, we can reject the null hypothesis with the critical level *α* if *F_F_* is greater than the corresponding critical value.

Three datasets are employed in our experiments; however, since the performance of each tested method is validated with GO annotations on both functional similarity and cellular co-localizations, each dataset yields two testing cases. Hence, we have four models, and six testing cases. For each testing case, we compute the average rank of each tested method based on their performance at each testing point. Note that the assessment and prediction are two different tasks. Hence, we conduct the corresponding tests separately.

Hence, in our experiments, *k* and *N* in (1) and (2) are 4 and 6, respectively. Hence, *F_F_* is distributed according to the *F*-distribution with 4-1 = 3 and (4-1)(6-1) = 15 degrees of freedom. The critical value of *F*(3, 15) for *α* = 0.05 is 3.29. Therefore, if the test scores of our experiments are greater than 3.29, we can reject the null hypothesis.

According to the performance of each tested algorithm in addressing the tasks of assessment and prediction as depicted in Figs. 1–6, we summarize their performance ranks in Tables I and II. Note that in each table, F.H. and C.C. stand for validating the performance of a tested algorithm with GO annotations of functional homogeneity and cellular co-localizations, respectively. Since IG cannot predict missing interactomes, we rank it behind the other algorithms that can do so. Then with the average rank of each algorithm, we compute the test scores as follows,
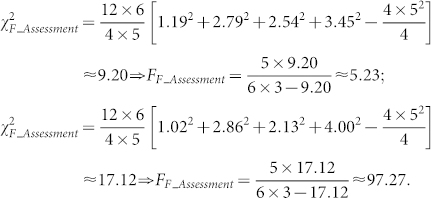
Both test scores are greater than 3.29. Hence, we conclude that the tested algorithms in our experiments are significantly different in performance with a confidence of 95%.

For further identifying the performance of tested algorithms, we employ the Nemenyi analysis^45^. In the test, two models are significantly different if the difference between their performance ranks is greater than the critical difference value^45^, which is given by
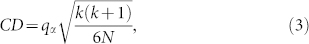
where *q_α_* is based on the Studentized range statistic^40^. With four models in the experiment, in our case the critical value *q_α_* = 2.291 with the critical level *α* = 0.1^45^. By substituting *k* = 4 and *N* = 6 along with *q_α_* into (3), we obtain that *CD* = 1.71, which indicates that any pair of models with a rank difference higher than 1.71 have significant difference in recommendation accuracy with a confidence of 90%.

**Figure 7:**
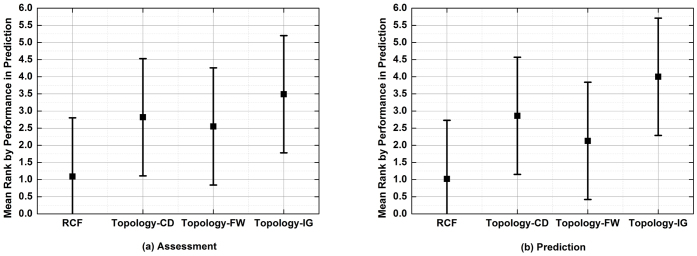
The results of Nemenyi analysis.

Fig. 7 depicts the results of the Nemenyi analysis. From Fig. 7, we see that from the statistical aspect, RCF outperforms CD and IG significantly. Although we cannot conclude that RCF significantly outperforms FW, the difference in their performance is still clear. Hence, based on the experimental results and significance analysis, we summarize that in comparison with three well-known and sophisticated topology-based algorithms, the proposed RCF achieved significantly higher performance in addressing the BIM problem on large, sparse HTS-PPI datasets.

## Discussion

From the Results Section, we see that the efficiency of the proposed RCF in addressing the BIM problem is supported by the experimental results. In this section, we discuss several related points.

### Basic assumption

Like the topology-based methods, the proposed CF-based framework also works based on the assumption that potential interactomes probably exist among proteins sharing many common interactive neighbors^23^,^24^,^25^,^26^,^27^,^40^,^41^. However, counter examples against this assumption can be found as concerned by pioneering researchers. Therefore, it is interesting to further study the cases where interactomes are supported by the common neighbors or not, to design some specific strategies. This will be our future work.

### Connections between CF-based and topology-based approaches

The main connection between these two kinds of approaches is their ability of addressing the BIM problem purely relying on HTS-PPI data, without the need of any additional information. However, they use different principles. Topology-based approaches explore of the neighborhood topology structures, based on which the connections among involved proteins are modeled^23^,^24^,^25^,^26^,^27^,^40^,^41^. For instance, CD works by solving the normalized difference between the direct neighbor sets of two proteins; FW further include the indirect associations among the neighborhoods of involved proteins for higher efficiency.

The proposed RCF, on the other hand, does not rely on such an exploration process. Its fundamental data source, i.e., the IW matrix, is built relying on the HTS-PPI data. Once obtaining this matrix, we treat it as the input data describing certain characteristics of involved proteins, without considering any topology information. The subsequent steps, i.e., feature extraction and mapping-indicator modeling, are carried out by manipulating the IW matrix. Its performance relies heavily on a mapping-indicator representing the relationship among involved proteins built on feature-vectors extracted from the IW matrix. As proven by the experimental results, a carefully designed mapping-indicator such as the I-Sim of RCC enables RCF to provide excellent performance in addressing the BIM problem.

### Possible extensions

In this work, we initialize the CF-based framework for the problem of BIM. The obtained RCF is a protein relationship-based model, which works by modeling the relationship among involved proteins based on given PPI data. However, as indicated by recent works in the area of recommender systems, such latent connections can also be obtained through optimization-based techniques like the EM-based methods or latent factor analysis^31^,^32^. These techniques can be also integrated into our framework to achieve highly efficient extensions. Meanwhile, in this work we only employ the HTS-PPI data as the input data. It will be interesting to see whether the better model can be achieved with the integration of more biological evidence. Such extensions will also be included in our future work.

## Methods

### A. The CF-based Framework for the BIM Problem

**Figure 8:**
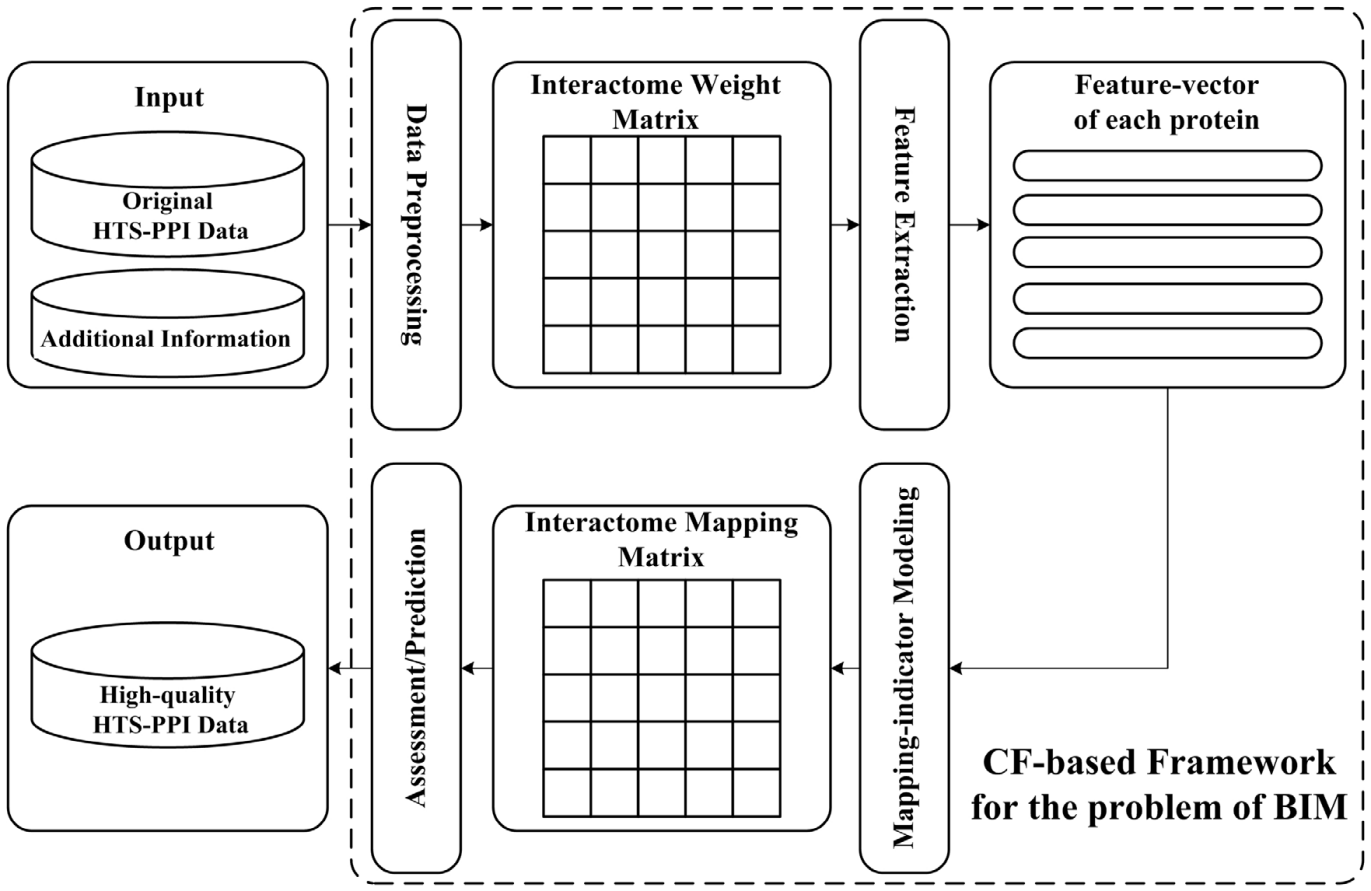
Framework of the CF-based approach to BIM.

Firstly, we present our CF framework for the BIM problem in Figure 8. As depicted in Figure 5, the proposed framework contains four steps, which are data preprocessing, feature extraction, mapping-indicator modeling and assessment/prediction, respectively. Next, we illustrate each step of our approach under this framework.

#### Data Preprocessing

When employing CF-based approaches to personalized-recommendation, we model the given data into a matrix that contains numerous missing entries. Its known entries are built based on their corresponding user-item usage history. With such a matrix, we build the patterns reflecting the relationship among involved users and items, thereby making reliable recommendations^29^,^30^,^31^,^32^. As mentioned before, BIM and personalized-recommendation have very similar solution spaces, where the key is to identify the connections among involved entities based on incomplete data. From this point of view, we adopt the idea of CF to transform the given HTS-PPI data along with other available information into an incomplete matrix as the data source. We define this matrix as follows,

#### Definition 1

Given a protein set *P*, an *interactome weight matrix Y* is a |*P*|×|*P*| matrix where each entry *y_i_*_,*j*_ corresponds to the interactome weight between proteins *i* and *j*.

Naturally, as the input data source, the construction of the interactome weight (IW) matrix has direct effect on the final output; therefore, it is vital to define its entries. In this work, we consider the following two factors regarding this issue,

- We focus on cases where only binary HTS-PPI data are available; hence, the IW value corresponding to each protein pair is set equal to the given HTS-PPI data, i.e., an IW value is equal to one if the corresponding HTS-PPI is given, and zero otherwise. Nonetheless, more specific IW settings can be employed when additional information, e.g., protein attributes^26^,^27^,^46^,^47^, is available; and
- Most interactomes are unknown in a sparse HTS-PPI network, and thus the corresponding IW matrix is very sparse with numerous zeroes. To decrease the distance among interacting proteins, a strategy commonly adopted by topology-based methods is to define the interactive neighbor set of a specified protein to include itself^21^,^25^,^26^,^27^. Here we set the diagonal entries of the IW matrix at one to achieve the same effect.

**Figure 9:**
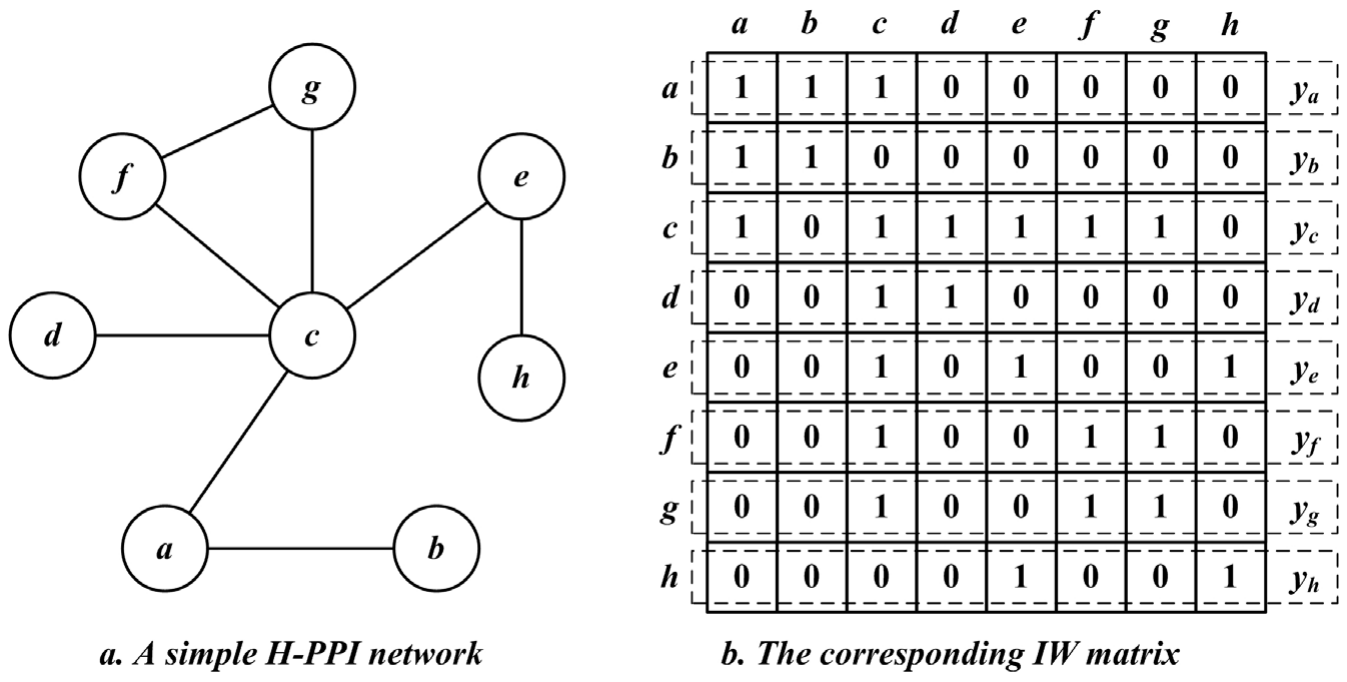
Illustrative example of an HTS-PPI network and corresponding IW matrix.

Based on the above inference, we set each *y_i_*_,*j*_ in *Y* according to the given HTS-PPI network *G* = (*V*, *E*) as follows,
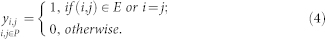
Note that binary HTS-PPI data are undirected; therefore, with (4) we obtain a symmetric IW matrix where each row/column represents the neighborhood of a specified protein in the given HTS-PPI network. The illustrative example of a simple network and the corresponding IW matrix is given in Figure 9.

#### Feature Extraction

Obviously, although an IW matrix *Y* is usually very sparse, it still contains rich information about the interactive neighborhood of those proteins involved in the HTS experiments. As indicated by research in the CF area^29^,^30^,^31^,^32^, given a sparse target matrix, it is feasible to model the relationship among involved entities, i.e., users/items, based on it. A straightforward but efficient way^29^,^30^,^31^ to do so is by treating each row/column vector as the feature-vector describing a specified user/item, and solving the corresponding vector similarity to model the desired relationship.

With the same principle, here we extract each row vector from an IW matrix as the feature-vector of the corresponding protein as depicted in Figure 6(b), to model the protein-protein relationship. In the BIM context, this straightforward strategy is also reasonable, since each row vector in the IW matrix describes information about the neighborhood of a specified protein directly. Nevertheless, more specific strategies regarding this issue need investigations.

#### Mapping-indicator Modeling

With the extracted IW feature vectors, we model the interactome mapping-indicator, which measures the likelihood of each interactome inferred from the HTS-PPI data. In this work, we build the mapping indicator with the inter-neighborhood similarity, which is defined as follows,

#### Definition 2

Given an interactome weight matrix *Y*, the *inter-neighborhood similarity* between proteins *i* and *j* is given by *sim_i,j_* : = *f*(*y_i_*,*y_j_*), where *y_i_* and *y_j_* denote the interactome weight feature-vectors for proteins *i* and *j* extracted from *Y*, and *f*(*y_i_*,*y_j_*) denotes a function of *y_i_* and *y_j_* to compute the vector-similarity between *y_i_* and *y_j_*.

Note that *f* (*y_i_*, *y_j_*) in the above definition can be defined differently and will be discussed later. With *Y* built on (4), the inter-neighborhood similarity (I-Sim) unveils how a given HTS-PPI is collaboratively supported by the interactive neighbors attached to the corresponding pair of proteins, and plays a critical role in our approach. In the next section, we will present a novel I-Sim metric which is especially designed for binary HTS-PPI data corresponding to sparse networks.

#### HTS-PPI Assessment/Prediction

Once we obtain the mapping-indicator matrix consisting of available mapping-indicators on each protein pair, we address the BIM problem as follows,

- Assessment. HTS-PPIs with high mapping-indicators are regarded as highly reliable, and vice versa; and
- Prediction. Missing interactomes corresponding to highest mapping-indicators are regarded to possess the highest probability to appear in nature.

In this work, the mapping-indicators are modeled by I-Sim among IW feature vectors. The intuition behind such a design is that the feature-vectors extracted from the IW matrix demonstrate the interactome characteristics of involved protein pairs, which reflect the process of the corresponding HTS experiments. Hence, I-Sim measuring the closeness of IW feature-vectors is able describe the likelihood of interactions among corresponding proteins.

### B. I-Sim Design

#### Benchmark

In this work, I-Sim directly decides the values of the mapping-indicators which represent the likelihoods of corresponding HTS-PPIs. Therefore, it is vital to design an efficient I-Sim metric, which is able to model the protein-protein relationship based on binary HTS-PPI data precisely, for achieving high performance. According to pioneering research^29^,^30^,^31^, a simple and basic choice is the cosine similarity. With it, we build the I-Sim between proteins *i* and *j* as follows, 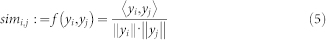
where 〈.,.〉 denotes the inner product between two vectors, and 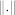 denotes the Euclid norm of the given vector. Note that based on (5), *sim_i_*_,*j*_ increases if proteins *i* and *j* have more common neighbors, i.e., the numerator 〈*y_i_*,*y_j_*〉 becomes larger; and decreases if either involved protein has many neighbors, i.e., the denominator 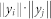 becomes larger.

#### Rescaled cosine coefficient

The cosine similarity (5) provides us with a benchmark to model the I-Sim among involved proteins. However, it suffers from instability, thereby resulting in low accuracy^29^,^30^,^31^. For instance, consider the I-Sim values corresponding to HTS-PPIs (*a*, *b*) and (*a*, *c*) in Figure 6(a); according to (5), we solve *c_a_*_,*b*_ and *c_a_*_,*c*_ with *y_a_*, *y_b_* and *y_c_* shown in Figure 6(b) as follows,
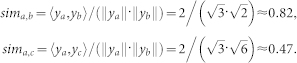
Note that judging from the network depicted in Figure 6(a), HTS-PPIs (*a*, *b*) and (*a*, *c*) are very close. However, with the cosine similarity, we arrive at the conclusion that the HTS-PPI (*a*, *b*) is far more reliable than (*a*, *c*). Hence, the HTS-PPI (*a*, *c*) may be identified as noise due to the dense neighborhood of protein *c*. As mentioned before, HTS-PPI data usually contain false-positive noises^10^,^11^,^12^,^13^,^14^. When dealing with the BIM problem on such noisy data, the instability of cosine similarity can result in both problems of false-negative and false-positive, i.e., actually reliable HTS-PPIs are assessed as unreliable, and impossible interactomes are predicted to exist. To alleviate such inefficiency, we propose the rescaled cosine coefficient (RCC), which integrates saturation-based strategies into the cosine similarity (5) for achieving more precise protein-protein relationship.

First, we integrate saturation factors into the denominator of (5) for controlling the impact of vector norms. As depicted in Figure 6, the norm of the IW feature-vector on a specified protein actually demonstrates the size of its neighborhood. With the integration of saturation factors, we intend to shrink the numerical gap among obtained I-Sim on extreme cases, as well as maintaining the relative order of I-Sim values supported by an equal number of common neighbors. Hence, we consider incorporating a constant saturation parameter *C_Y_* into the denominator of (5), to obtain:
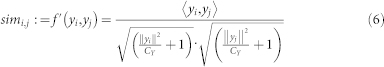
With (6), the denominator is transformed into a saturation function of 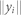 and 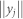 which possesses the following characteristics,

- The same as the denominator of the cosine similarity, it is monotonously non-decreasing with 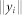 and 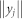; hence, the relative order of I-Sim values supported by an equal number of common neighbors remains; and
- The numerical differences caused by extremely large/small norms of IW feature-vectors are shrunk; hence, the impact by noise data is controlled.

For instance, by setting *C_Y_* = 20, we solve the I-Sim values *sim_a_*_,*b*_ and *sim_a_*_,*c*_ in the previous case according to (6) as follows,
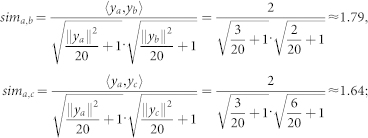
where we see that the numerical gap between *sim_a_*_,*b*_ and *sim_a_*_,*c*_ is greatly shrunk, and their relative order remains. Note that with the incorporation of *C_Y_*, the value of each I-Sim is enlarged. Nonetheless, since we mainly care about the ranking of interactomes by I-Sim, such numerical value changes will not affect the process of accurate assessment/prediction.

Meanwhile, the inner product 〈*y_i_*,*y_j_*〉 in (5) and (6) actually describes how the I-Sim between proteins *i* and *j* is collaboratively supported by their common neighbors. Note that when addressing the BIM problem, extreme cases, i.e., HTS-PPIs supported by many or few common neighbors, are easy to distinguish. However, it is relatively hard to distinguish those supported by close numbers of common neighbors due to the possible existence of noise data. To well handle them, we further introduce a saturation factor into the numerator of (6) to

- Improve the sensitivity of I-Sim in distinguishing HTS-PPIs supported by frequent numbers of common neighbors; and
- Reduce the impact of noises by integrating the prior knowledge describing the global distribution of the given data.

We first introduce the inter-neighborhood similarity support defined next.

#### Definition 3

Given an interactome weight matrix *Y*, the *inter-neighborhood similarity support* on protein pair (*i, j*) is the number of common neighbors supporting the inter-neighborhood similarity between *i* and *j*, and given by *n_i,j_* = 〈*y_i_*,*y_j_*〉.

Actually, *n_i_*_,*j*_ supports the I-Sim between proteins *i* and *j* just like interpersonal relationship; people cannot judge the relationship between each other based on few contacts only, and vice versa. From this point of view, it is reasonable to enlarge I-Sim values with a rescaling coefficient relying on the corresponding I-Sim supports for demonstrating their strong confidence, and the main concern turns to the design of this rescaling coefficient.

A straightforward solution to this problem is the max-min normalization, i.e., set the rescaling coefficient *r_i_*_,*j*_ corresponding to *n_i_*_,*j*_ as *r_i_*_,*j*_ = *n_i_*_,*j*_/*n*_max_. However, as mentioned before, HTS-PPI data usually contains noise data, which impacts the performance of this strategy. Nonetheless, with the given dataset large enough, it is reasonable to fit all observed I-Sim supports with a normal distribution, of which the average and variation are estimated as follows,
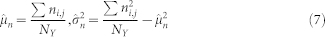
Note that *N_Y_* in (7) denotes the number of non-zero neighborhood similarity-supports from the IW matrix *Y*. With 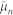 and 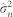 we estimate the probability that *n_i_*_,*j*_ is greater than or equal to the others as the rescaling coefficient for each neighborhood similarity:
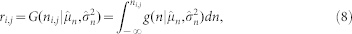
where 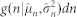 denotes the density function of the normal distribution with 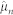 and 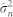. With this strategy, we actually introduce the prior knowledge describing the distribution of I-Sim supports on the whole HTS-PPI dataset, into the rescaling coefficient supporting the confidence of each single I-Sim. With such a design we can reduce the impact of noise data, thereby improving the performance.

One concern with (8) is the high complexity to solve the integral. However, this can be addressed through the Taylor approximation. Note that the cumulative distribution function (8) also possesses the characteristic of saturation, i.e., the generated rescaling coefficients are sensitive in distinguishing I-Sim with close I-Sim supports. By incorporating (8) into (6), we obtain
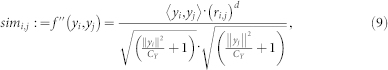
where *d* decides the rescaling effect brought by the rescaling coefficient *r_i_*_,*j*_. Note that according to (8), all rescaling coefficients lie in the scale of (0, 1); therefore, the value of *sim_i_*_,*j*_ decreases as *d* increases. However, as *d* increases, the relative gap among I-Sim values depending on rescaling coefficients also increases, thereby enlarging their effect. If *d* = 0, the obtained I-Sim values are not affected by rescaling coefficients.

Based on the above analysis, let *rcc_i,j_* : = *sim_i,j_* : = *f″*(*y_i_*,*y_j_*), we propose RCC as follows,
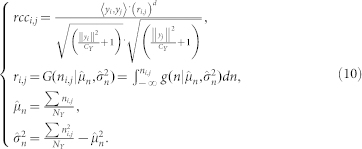


## Author Contributions

X.L., Z.Y. and M.Z. wrote the main manuscript text; X.L., Z.Y., S.L. and H.L. did the experiment; S.L., H.L., Y.X. and Q.Z. helped to justify the theory; all authors reviewed the manuscript.

## Supplementary Material

Supplementary Information Hyper parameter-sensitive tests for RCF on experimental datasets

## Figures and Tables

**Table 1:** The rank of tested methods by their performance in assessment

	D1		D2		D3		
Method	F.H.	C.C.	F.H.	C.C.	F.H.	C.C.	Avg.
RCF	1.07	1.02	1.02	1.07	1.11	1.27	1.09
CD	2.42	3.11	2.97	2.73	2.80	2.87	2.82
FW	2.76	2.6	2.11	2.36	3.27	2.20	2.55
IG	3.71	3.24	3.89	3.84	2.78	3.47	3.49

**Table 2:** The rank of tested methods by their performance in prediction

	D1		D2		D3		
Method	F.H.	C.C.	F.H.	C.C.	F.H.	C.C.	Avg.
RCF	1.00	1.00	1.00	1.00	1.00	1.10	1.02
CD	3.00	3.00	3.00	3.00	2.85	2.30	2.86
FW	2.00	2.00	2.00	2.00	2.15	2.60	2.13
IG	4.00	4.00	4.00	4.00	4.00	4.00	4.00
