# Supplementary material for: A Highly Efficient Approach to Protein Interactome Mapping Based on Collaborative Filtering Framework
Source: Sci Rep. 2015 Jan 9;5:7702. doi: 10.1038/srep07702 (PMC4287731; doi:10.1038/srep07702)
Supplement: Supplementary Information — Hyper parameter-sensitive tests for RCF on experimental datasets [file srep07702-s1.pdf]

A Highly Efficient Approach to Protein Interactome Mapping Based on Collaborative Filtering Framework

Xin Luo, Zhuhong You, Mengchu Zhou, Shuai Li, Hareton Leung, Yunni Xia, and Qingsheng Zhu

Supplementary Information: Hyper parameter-sensitive tests for RCF on experimental datasets

Note that RCF employs two hyper-parameters, i.e., C_Y and d, to control the rescaling process. Obviously, these two parameters will affect the final output. Hence, it is necessary to conduct parameter-sensitive tests with respect to them. We first fix C_Y=0, and test the performance of RCF with d ranging in [0, 100] to check the effect brought by rescaling coefficients relying on I-Sim supports. Then we fix d=1, and test RCF with C_Y ranging in [0, 600] to see its effect. During such tests, the performance of RCF in HTS-PPI assessment and prediction on both datasets is recorded.

Assessment. Figures A1-A2 depict the performance of RCF in assessing the reliability of HTS-PPIs on both datasets with d varying. As shown in Figures A1-A2, d has critical impact on the performance of RCF in HTS-PPI assessment. On D1, with d=10, about 77.0% of the top 50% of HTS-PPIs selected by RCF are supported by functional homogeneity, and about 78.4% of them are supported by cellular co-localization; however, with d = 0, these two ratios reduce to about 70.6% and 73.3%, respectively, as shown in Figures A1(a) and A1(c). Similar phenomena can be also observed during the test on D2, as shown in Figure A2(a) and A2(c).

During our tests, RCF tends to obtain stronger correlation with function homogeneity and localization coherence a d increases at the very beginning; once d reaches a certain threshold, the outputs of RCF stabilize. This can be seen more clearly from the average values of the correlation with function homogeneity and localization coherence by RCF, as depicted in (b) and (d) of Figures A1-A2. For both datasets, the correlation with function homogeneity and localization coherence of RCF stabilizes with d bigger than 20.

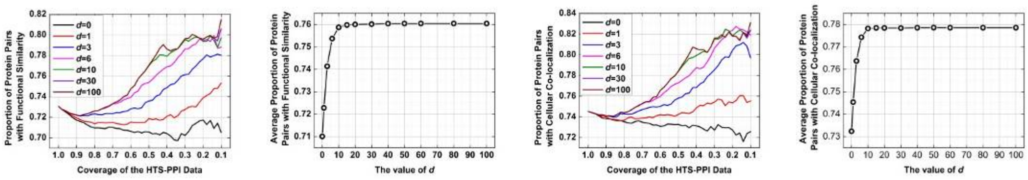

(a) Functional homogeneity **(b) Average F-homogeneity** **(c) Localization coherence** **(d) Average L-coherence**

Figure A1. Performance of RCF in assessment with C_Y=0 and d changing on D1.

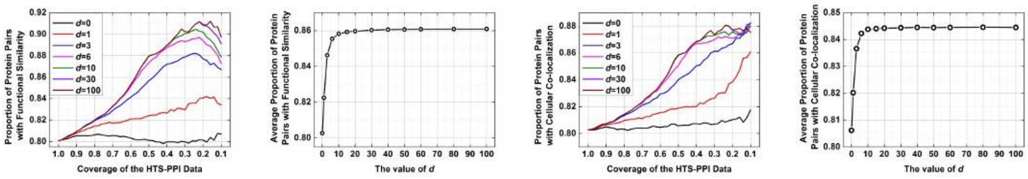

(a) Functional homogeneity **(b) Average F-homogeneity** **(c) Localization coherence** **(d) Average L-coherence**

Figure A2. Performance of RCF in assessment with C_Y=0 and d changing on D2.

Figures A3-A4 depict the performance of RCF in the task of HTS-PPI assessment on both datasets with C_Y changing. From these results, we see that the performance of RCF also relies on the value of C_Y. As shown in Figures A4(a) and A4(c), by setting C_Y=600 on D2, about 87.9% of the top 50% of HTS-PPIs selected by RCF are supported by functional similarity, and about 85.8% of them are supported by localization coherence. However, with C_Y=0, these two ratios reduce to about 82.4% and 81.9%, respectively. Similar phenomena can be observed in the tests on D1, as shown in Figure A3.

Meanwhile, the performance of RCF also stabilizes when C_Y gets large enough. On both datasets, the correlation with functional homogeneity and localization coherence of RCF stabilizes with C_Y bigger than 150, as shown in (b) and (d) of Figures A3-A4.

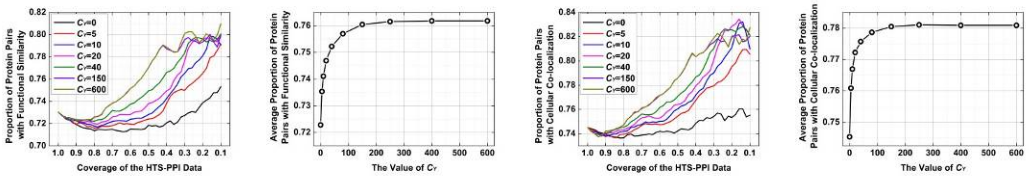

(a) Functional homogeneity (b) Average F-homogeneity (c) Localization coherence (d) Average L-coherence

Figure A3. Performance of RCF in assessment with d=1 and C_Y changing on D1.

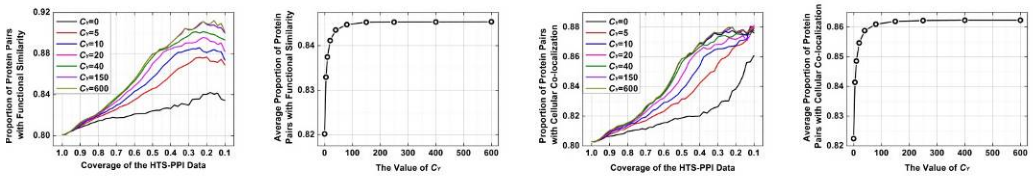

(a) Functional homogeneity (b) Average F-homogeneity (c) Localization coherence (d) Average L-coherence

Figure A4. Performance of RCF in assessment with d=1 and C_Y changing on D2.

To summarize, when dealing with the task of assessment, the performance of RCF relies on d and C_Y. In general, when they are large enough, RCF achieves fine performance in assessing the reliability of HTS-PPIs.

Prediction. Figures A5-A6 depict the performance of RCF when predicting missing interactomes with d changing. From these two figures, we see that when processing the task of prediction, d plays an important role in affecting the performance of RCF. Generally, its prediction accuracy increases with d increasing. As shown in Figures A5(a) and A5(c), with d=100 on D2, about 42.5% HTS-PPIs of the 20,000 interactomes predicted by RCF are supported by functional similarity, and about 64.4% of them are supported by localization coherence; however, with d=0, these two ratios drop to about 22.5% and 39.6%, respectively. Similar situations can also be found in the experiments on D2, as shown in Figures A6(a) and A6(c). Generally, with d large enough, the prediction accuracy of RCF tend to stabilize, as shown in (b) and (d) of Figures A5- A6.

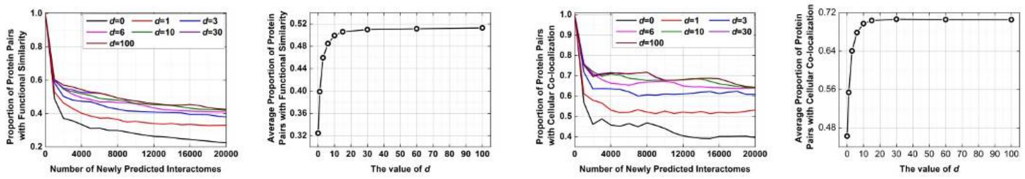

(a) Functional homogeneity (b) Average F-homogeneity (c) Localization coherence (d) Average L-coherence

Figure A5. Performance of RCF in prediction with C_Y=0 and d changing on D1.

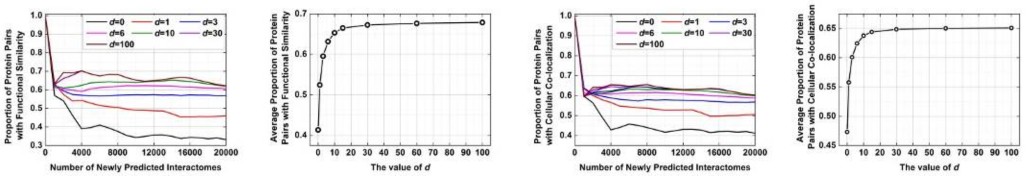

(a) Functional homogeneity (b) Average F-homogeneity (c) Localization coherence (d) Average L-coherence

Figure A6. Performance of RCF in prediction with C_Y=0 and d changing on D2.

Figures A7-A8 depict prediction accuracy of RCF on both datasets with C_Y varying. From these results, C_Y also affects the prediction accuracy of RCF. On Both datasets, with C_Y>250, the prediction accuracy stabilizes, as shown in Figures A7-A8. To summarize, the predicting accuracy of RCF relies

on large values *d* and C_Y.

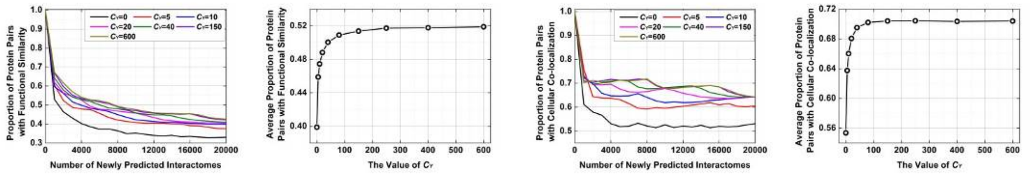

(a) Functional homogeneity **(b) Average F-homogeneity** **(c) Localization coherence** **(d) Average L-coherence**

Figure A7. Performance of RCF in prediction with d=1 and C_Y changing on D1.

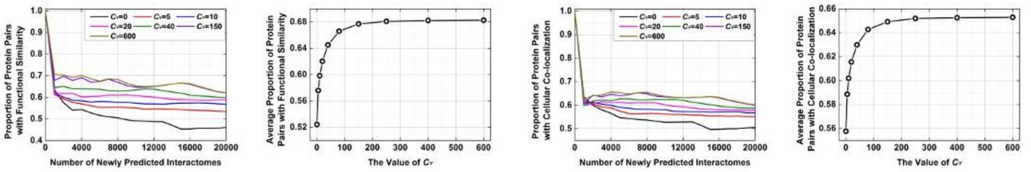

(a) Functional homogeneity **(b) Average F-homogeneity** **(c) Localization coherence** **(d) Average L-coherence**

Figure A8. Performance of RCF in prediction with d=1 and C_Y changing on D2.
